# Unusual reticulin staining pattern in well-differentiated hepatocellular carcinoma

**DOI:** 10.1186/1746-1596-6-15

**Published:** 2011-02-22

**Authors:** Heng Hong, Bryan Patonay, James Finley

**Affiliations:** 1 Department of Pathology and Laboratory Medicine, Brody School of Medicine at East Carolina University, 600 Moye Boulevard 7S10, Greenville, North Carolina 27858, USA

## Abstract

**Background:**

Special stains, such as reticulin stain and CD34 immunostain, are very helpful in the diagnosis of well differentiated hepatocellular carcinoma (HCC). Most studies have shown that absent or decreased reticulin stain or an abnormal reticulin pattern with widened trabeculae is reliable for the diagnosis of well-differentiated HCC.

**Case report:**

We report here two cases of well differentiated HCC with an unusual reticulin staining pattern. A strongly positive reticulin network was preserved within the tumor, which surrounded individual tumor cells in a monolayered trabecular pattern. At the same time, an increased CD34 stain was present in the tumor.

**Conclusions:**

This unusual reticulin pattern represents part of the diverse reticulin staining patterns seen in HCC. Although this staining pattern is rare, it should be recognized when diagnosing well-differentiated HCC in small samples such as cellblock of fine needle aspiration or small core biopsies.

## Background

Image guided fine needle aspiration (FNA) and core biopsy of the liver have become a crucial part of the clinical evaluation and management of massive liver lesions [1]. Because of the improvement in imaging techniques, more small hepatic lesions can be identified, and surgical pathologists are now faced with an increased number of liver FNA and core biopsies. The diagnosis of moderately to poorly differentiated hepatocellular carcinoma (HCC) normally is not difficult for experienced pathologists, but the diagnosis of well-differentiated HCC by FNA and core biopsy can be very challenging. Use of reticulin stain and other special studies has been found to be helpful in the differential diagnosis between well-differentiated HCC and benign hepatic nodules [2,3]. Abnormal reticulin stain patterns, either decreased reticulin stain or widened trabeculae with greater than three cell layers in thickness, are considered to be reliable for the diagnosis of a well-differentiated HCC [4,5]. However, unusual reticulin staining patterns can occasionally be encountered in HCC, and make the diagnosis very difficult. Here we describe two cases of well-differentiated HCC with an unusual reticulin staining pattern in their primary biopsies.

## Case presentation

**Figure 1:**
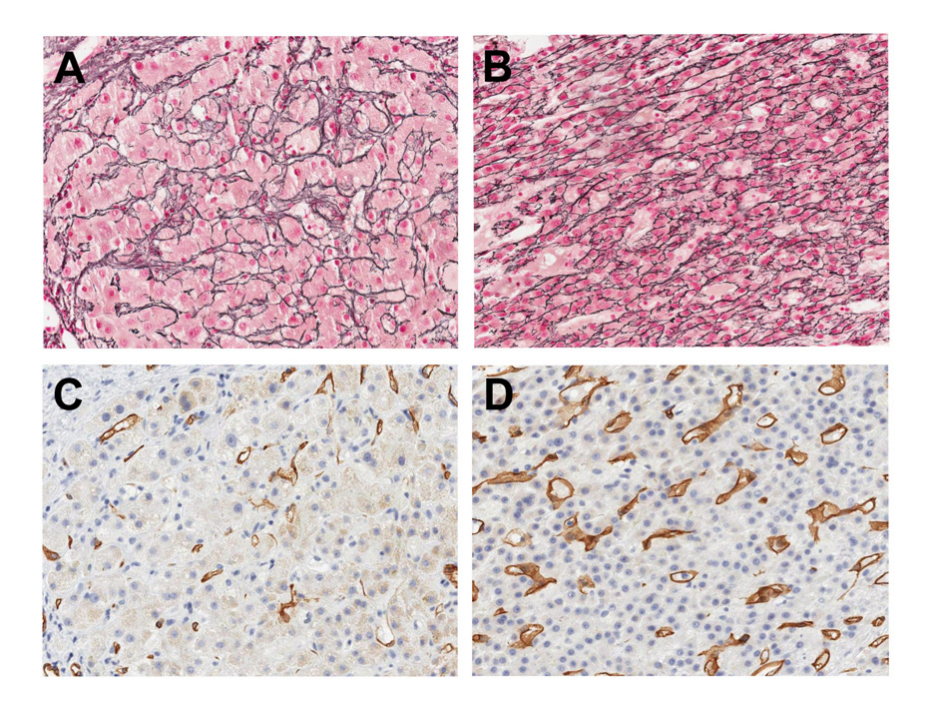
**Reticulin and CD34 stains for the liver core biopsy of a 55-year old patient with history of cirrhosis and well differentiated hepatocellular carcinoma (HCC)**. (A) Reticulin stain in the area of cirrhosis. Well preserved reticulin network is present. The trabecular plates are thin, and less than two cell in thickness. (B) Reticulin stain in the area of HCC. The tumor shows well preserved reticulin network, which surrounds individual tumor cells in a monolayered trabecular pattern. (C) CD34 stain in the area of cirrhosis (same area as A). Only rare positive stain is present in the peripheral area of the regenerative nodules. (D) CD34 stain in the area of HCC (same area as B). Diffuse positive sinusoidal CD34 stain is present in the tumor.

The first patient was a 55-year old male with a history of alcohol abuse and cirrhosis. He presented with an 8.4-cm liver mass which was identified in ultrasound examination. Ultrasound also confirmed the presence of cirrhosis and ascites. Laboratory results showed abnormal liver function tests and a blood alpha fetoprotein level of 767 ng/ml. Ultrasound-guided FNA and core biopsy were performed on his liver mass. The smear slides of FNA showed many mildly atypical hepatocytes, with numerous stripped atypical hepatocytic nuclei. The concurrent liver core biopsy demonstrated that besides the presence of cirrhosis (Figure 1, A &1C), there were nodules composed of uniform atypical hepatocytes with consistently high nuclear-to-cytoplasmic ratios, which were morphologically consistent with well differentiated HCC (Figure 1, B &1D). In immunohistochemical stains, a positive sinusoidal CD34 stain was diffusely present in areas of tumor (Figure 1, D), but only focally and weakly present in the area of cirrhosis (Figure 1, C). On reticulin stain, a normal reticulin staining pattern was observed in cirrhotic area which showed preserved thin (one or two cells) hepatic trabeculae (Figure 1, A). In the area of tumor, a strongly positive reticulin network was present, which surrounded individual tumor cells in a monolayered trabecular pattern. No thickened hepatocytic trabeculae were seen in the tumor (Figure 1, B). This patient suffered fulminant liver failure later and died nine days after the biopsy.

**Figure 2:**
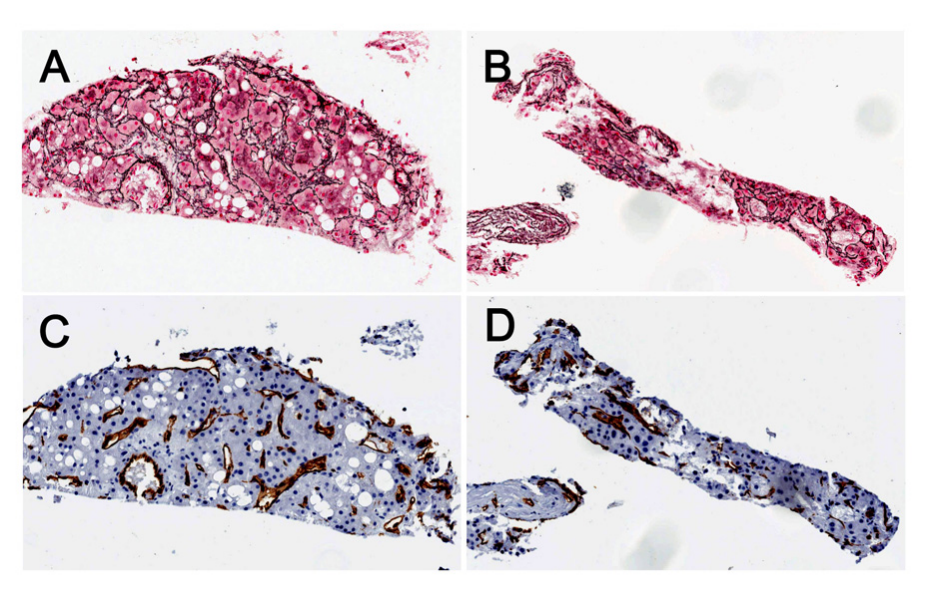
**Reticulin and CD34 stains for the liver core biopsy of a 71-year old patient with well differentiated hepatocellular carcinoma (HCC)**. (A) Abnormal reticulin staining pattern in one area of HCC, showing widened trabeculae with greater than three cell layers in thickness. (B) Unusual reticulin staining pattern in another area of HCC. The tumor shows well preserved reticulin network, which surrounds individual tumor cells in a monolayered trabecular pattern. (C) CD34 stain in HCC (same area as A). Diffuse positive sinusoidal CD34 stain is present. (D) CD34 stain in HCC (same area as B). Diffuse positive sinusoidal CD34 stain is present.

The second patient was a 71-year old male with a history of prostate cancer and presented to the hospital with abdominal pain. CT scan showed multiple liver masses and diffuse pulmonary metastases. Ultrasound guided FNA and core biopsy of the liver mass were performed. The smear slides from the liver FNA showed many hepatocytes which were cytologically bland. In the concurrent liver core biopsy, a well-differentiated HCC was identified. Most areas of the tumor showed a "typical" thickened hepatocytic trabecular pattern in reticulin stain (Figure 2, A) and diffuse positive sinusoidal CD34 pattern on immunostain (Figure 2, C). In some areas of the tumor, however, a well preserved reticulin network was still present (Figure 2, B), with features similar to what were described in the first patient. Immunostain for CD34 was diffusely positive in this area of tumor (Figure 2, D). This patient passed away one week after the biopsy was done.

## Discussion

FNA and needle core biopsy are commonly used in the diagnosis of HCC, however, the distinction between well-differentiated HCC and benign nodular lesions of the liver can be very difficult [6]. Use of reticulin stain and other special studies has been found to be helpful in the differential diagnosis between well-differentiated HCC and benign liver lesions [2,3]. While normal liver tissue and most benign hepatic lesions show a well preserved reticulin network, most published reports suggest that well-differentiated HCC has either absent or decreased reticulin, or an abnormal reticulin staining pattern with widened trabeculae [4,5]. Ferrell et al. found that loss of reticulin stain, together with other architectural features, was very helpful in differentiating benign macroregenerative nodules from HCC [7]. Bergman et al. studied reticulin staining in cell blocks of FNA for the diagnosis of HCC, and found that all the HCC showed an abnormal reticulin pattern, which they defined as: virtually absent, decreased or variable reticulin, or thickened trabeculae greater than three cell layers [5]. Although this definition was widely accepted in the diagnosis of well differentiated HCC, rare cases of unusual reticulin networks in HCC have also been reported. Wilkens et al. described a case of well-differentiated HCC which showed chromosomal aneuploidy, but the reticulin network was well preserved [8]. For many pathologists, however, the presence of a preserved reticulin network may still lead to uncertainty in the diagnosis of well-differentiated HCC.

Immunohistochemical stain for CD34 is another commonly used special study in the diagnosis of well-differentiated HCC [2]. In 1997 Tanigawa et al. reported that CD34 immunostaining was confined to portal tracts and to a few periportal sinusoids in normal liver tissue, but in HCC an intense sinusoidal staining pattern for CD34 was present [9]. In a study performed by de Boer et al. [10], the use of CD34 and reticulin stain in the diagnosis of HCC was compared. While CD34 showed diffuse positive staining in HCC but not in normal liver tissue, positive staining in some cases of benign hepatic lesions, such as focal nodular hyperplasia and adenoma, was also noted [10]. de Boer et al. found that reticulin stain more consistently distinguished between benign and malignant hepatocellular lesions than CD34 immunostain. It is believed that a proper diagnosis of well-differentiated HCC should be made by correlating clinical and imaging findings, laboratory tests, morphological features of biopsy, and special studies such as reticulin and CD34 stains.

For the two patients discussed in this report, their clinical presentations, the histological features of liver biopsies and the diffusely positive CD34 immunostain are consistent with a diagnosis of well-differentiated HCC. However, the monolayered trabecular pattern of reticulin staining was an unusual finding and made the diagnoses more difficult. After carefully observing the preserved reticulin network in these two cases, we found that the preserved reticulin network in HCC tends to surround individual hepatocytes, and this pattern is somewhat different from the well formed thin trabecular plates in benign liver tissue. In the biopsy of the second patient, many areas of the HCC still showed the "typical" abnormal reticulin stain pattern which fits the description by Bergman et al. (Figure 2, A), but in some other areas, HCC showed the preserved reticulin network (Figure 2, B). This finding suggests that HCC may have diverse reticulin patterns in different portions of the tumor. In a small specimen, such as core biopsy or cell block made from FNA material, if only the portion of tumor with well preserved reticulin network is present, the diagnosis can be challenging. In summary, we believe it is important to recognize the presence of different reticulin staining patterns in the evaluation of small biopsies for the diagnosis of HCC. The diagnosis of well differentiated HCC in small biopsies should be made in correlation with clinical history, imaging study, serology, histologic and cytologic features and other special studies such as reticulin stain and CD34 immunostain.

## Conclusions

Although abnormal reticulin stain patterns, either decreased reticulin stain or widened trabeculae, are considered to be reliable for the diagnosis of a well-differentiated HCC, occasional unusual reticulin stain patterns can be encountered. The preserved reticulin staining in HCC described in this report represents part of the diverse reticulin staining patterns in the tumor. Although this preserved reticulin staining pattern is rare, it should be recognized when establishing a diagnosis of well-differentiated HCC in small samples such as cellblocks or small core biopsies.

## Consent

This case report was based on the existing data, and the patients' identification was kept confidential in this study. This case report does not meet definition of human subject research by University and Medical Center Institutional Review Board of East Carolina University, and no consent form was obtained for this study.

## Competing interests

The authors declare that they have no competing interests.

## Authors' contributions

All authors (HH, BP and JF) have made substantial contributions to conception and design, data collection and interpretation, literature search, and manuscript preparation. All authors read and approved the final manuscript.
